# Anomalous Enhancement of Mechanical Properties in the Ammonia Adsorbed Defective Graphene

**DOI:** 10.1038/srep33810

**Published:** 2016-09-26

**Authors:** Fengxian Ma, Yalong Jiao, Yuantong Gu, Ante Bilic, Ying Chen, Zhongfang Chen, Aijun Du

**Affiliations:** 1 School of Chemistry, Physics and Mechanical Engineering, Queensland University of Technology, Gardens Point Campus, QLD 4001, Brisbane, Australia; 2 CSIRO Data61, Molecular and Materials Modelling, Docklands 3008 VIC, Australia; 3 Institute for Frontier Materials, Deakin University, Waurn Ponds, VIC 3216, Australia; 4 Department of Chemistry, Institute for Functional Nanomaterials, University of Puerto Rico, San Juan, Puerto Rico 00931, United States

## Abstract

Pure graphene is known as the strongest material ever discovered. However, the unavoidable defect formation in the fabrication process renders the strength of defective graphene much lower (~14%) than that of its perfect counterpart. By means of density functional theory computations, we systematically explored the effect of gas molecules (H_2_, N_2_, NH_3_, CO, CO_2_ and O_2_) adsorption on the mechanical strength of perfect/defective graphene. The NH_3_ molecule is found to play a dominant role in enhancing the strength of defective graphene by up to ~15.6%, while other gas molecules decrease the strength of graphene with varying degrees. The remarkable strength enhancement can be interpreted by the decomposition of NH_3_, which saturates the dangling bond and leads to charge redistribution at the defect site. The present work provides basic information for the mechanical failure of gas-adsorbed graphene and guidance for manufacturing graphene-based electromechanical devices.

Graphene, as a single-layer of *sp*^2^-hybridized carbon atoms arranged in a honeycomb lattice, has demonstrated many unique physical and chemical properties^1^. In particular, pristine graphene possesses exceptionally high stiffness and strength, and is known as the strongest two-dimensional material, which inspires the explorations of a wide range of potential applications such as lightweight and high strength materials and composites^2^,^3^,^4^,^5^,^6^,^7^. However, the existing experiments^8^,^9^,^10^ reported rather different values of in-plane stiffness for graphene, partly due to the different experimental conditions. Specifically, the emergence of defects in the graphene (D-graphene) lattice is ubiquitous, due to fabrication processes^11^,^12^,^13^ and the environmental conditions to which the graphene sheets are exposed^14^,^15^,^16^. Such defects would inevitably degrade the ideal strength of graphene^17^, resulting in the reduction of its breaking strength to only ~14%. In this context, how to improve the stiffness of D-graphene is of great importance for its applications in graphene-based nanoelectromechanical devices. Moreover, the presence of defects is also essential for the desired functionality, for example, for the applications in biodevices and DNA-decorated graphene^18^,^19^.

Noticeably gas adsorption on nanomaterials^20^,^21^,^22^,^23^,^24^,^25^,^26^,^27^ is attracting great interest because provides an effective method to control the spatial organization of adsorbates that are known to modify the chemical and electronic properties of the host materials. For example, Schedin *et al*.^28^ experimentally demonstrated that micrometre-size sensors made from graphene can detect individual gas molecules adsorbed on graphene. Inspired by this exciting experimental finding, Leenaerts *et al*.^29^ studied the optimal adsorption position and orientation of NH_3_, CO, NO_2_ and NO on a graphene substrate by means of density functional theory (DFT) computations, and pointed out that the dramatic change of the electronic properties after the adsorption of the gas molecule contributes to the high performance of graphene sensors. So far, a considerable number of theoretical and experimental studies have been performed to investigate the effect of adsorbates on the structural and electronic properties of host materials. However, the effect of gas adsorption on the mechanical properties has been rarely studied. Unveiling the effect of gas molecule adsorption on the mechanical properties of the graphene layer would help us understand this issue, which is important for its applications in flexible electronic nanodevices and other related fields.

In this paper, we performed a systematic study of gas adsorption on the perfect/defective graphene layer to evaluate the mechanical strength change. Many common gases, namely H_2_, N_2_, NH_3_, CO, CO_2_ and O_2_, have been considered due to their importance to environmental and industrial applications. We first investigate the structural and mechanical properties of the perfect graphene (P-graphene) with adsorbed gas molecules. Then, the change of ideal strain for the D-graphene upon gas molecule adsorption is investigated. Finally, the mechanisms behind the strength change are analysed to gain more insights into the effects of adsorbed molecules on the mechanical properties of D-graphene.

## Computational Method

Our spin polarized DFT computations were performed by utilizing Viena *ab intio* simulation package (VASP) code^30^,^31^ with the implemented projector augment wave (PAW) method^32^,^33^. The generalized gradient approximation in the Perdew-Burke-Ernzerhof form (GGA-PBE)^34^ was used to describe the exchange-correlation for electrons. A dispersion correction to the total energy (DFT-D3 method)^35^ was employed to simulate the long-range van der Waals interaction. The plane-wave energy cutoff was set to 400 eV for geometry optimization and to 500 eV for static electronic structure calculations. To study two-dimensional (2D) systems under the periodic boundary conditions, a vacuum layer with a thickness of at least 20 Å was used to avoid the interaction between periodic images. All the geometry structures were fully relaxed until energy and force were converged to 1.0^−5 ^eV and 0.005 eV/Å, respectively. A 5 × 5 × 1 and 17 × 17 × 1 Monkhorst–Pack k-point sampling was used for geometry optimizations and static electronic structure calculations, respectively. The adsorption energy (E_ad_) of a gas molecule on graphene layer was obtained by the following equation: *E*_ad_ = *E*_(gas+G)_ − (*E*_G_ + *E*_gas_), where *E*_(gas+G)_, *E*_G_ and *E*_gas_ are the total energies of the gas adsorbed graphene system, graphene and the adsorbed gas molecule, respectively. According to this definition, a more negative *E*_ad_ value indicates a more favourable adsorption.

The strain^36^,^37^ was added by changing the lattice parameters *a*, which is determined by the expression *a* = *a*_*0*_ (1 + ε). Here, *a*_*0*_ is the equilibrium lattice constants of graphene at 0% strain. The values of ε increased in steps of 0.4% until the graphene sheet fractured and the strain-stress relation was obtained. To eliminate the artificial effect of the out-of-plane thickness of the simulation box on the stress, we employed the second Piola–Kirchhoff stress^37^,^38^ to express the 2D forces with units of N/m. In order to lift the constraints imposed by periodic boundary conditions^39^, a 3 × 3 supercell was used for all the calculations. Note that only a single vacancy in each supercell was considered in order to reduce the complexity.

## Results and Discussion

### Structural and mechanical properties of the perfect graphene with adsorbed gas molecules

First, we search for the lowest-energy configuration when a gas molecule is adsorbed on P-graphene. To find the most favourable position for gas adsorption, we place the single gas molecule above the graphene layer at different distances and orientations. After full optimization, the obtained configurations are compared, and the energetically most favourable states are selected for further discussions.

**Figure 1:**
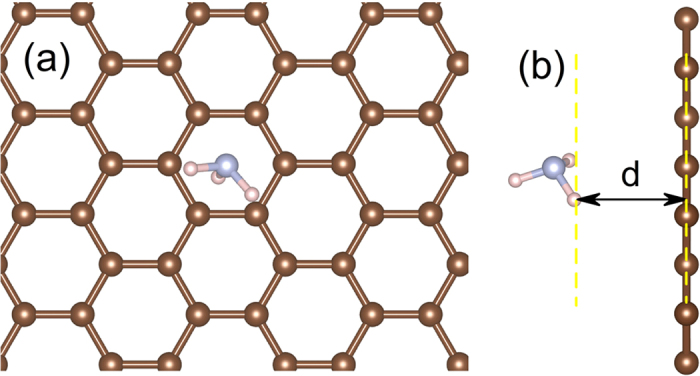
(**a**) Top and (**b**) side view of the relaxed structural model of gas (NH_3_) adsorption on the perfect graphene monolayer.

As a representative, the case for NH_3_ is presented in Fig. 1. The NH_3_ molecule prefers to locate in the hexagon center (Fig. 1a), and the calculated distance (see Table 1) between the NH_3_ molecules and graphene is 2.84 Å. The computed adsorbent-graphene distances, adsorption energies, and magnetic moments for other gas molecules (H_2_, N_2_, CO, CO_2_ and O_2_) are given in the Table 1. In the equilibrium, all these gas molecules are physically adsorbed on graphene with a distance between 2.68–3.32 Å. However, only the adsorption of H_2_, NH_3_ and O_2_ is exothermic while others are not favoured energetically, and only O_2_ adsorbed system is magnetic.

**Figure 2:**
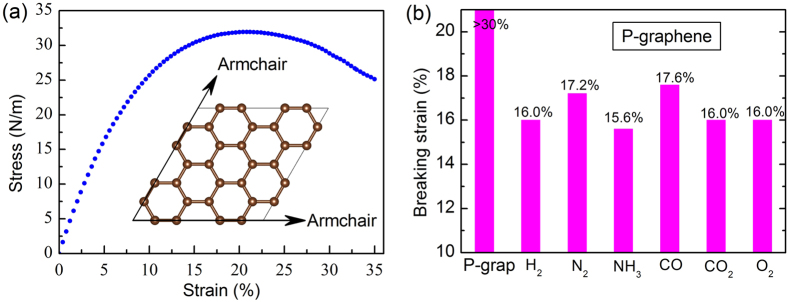
(**a**) The strain-stress curve for P-graphene. (**b**) The breaking strain for P-graphene adsorbed by H_2_, N_2_, NH_3_, CO, CO_2_ or O_2_ molecule.

Before studying the strain effect on the gas-adsorbed graphene, we investigated the strain-stress relationship in the P-graphene as shown in the Fig. 2a. In an attempt to evaluate the “minimum” ideal strain for graphene, both armchair and zigzag directions seem possible to be chosen. However, a previous report^40^ revealed that the armchair direction possesses lower ideal strain than the zigzag direction, since it is parallel to the carbon-carbon bond which dominates the mechanics of graphene^41^. Therefore, only the armchair direction is considered in our calculations. Our computations show that the P-graphene sheet can sustain a maximum stress up to ~32 N/m and its structure does not fracture even when the strain exceeds 30% (Fig. 2b), which is in consistent with previous theoretical work^40^.

However, the adsorption of a single gas molecule on the top of graphene sheet can significantly decrease its mechanical strength (Fig. 2b). The largest reduction derives from the adsorption of NH_3_, where the fracture point drops to only 15.6% relative to that of P-graphene (>30% of the fracture point). These results well explain why the synthesized graphene layers exhibit relatively low intrinsic strength (~15% of the fracture point) under realistic experimental conditions, which include gas exposure.

### Structural and mechanical properties of the defective graphene with adsorbed gas molecules

**Figure 3:**
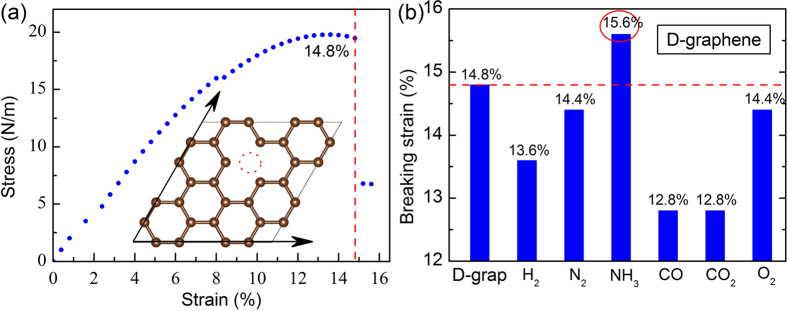
(**a**) The strain-stress curve for D-graphene. (**b**) The breaking strain for D-graphene adsorbed by H_2_, N_2_, NH_3_, CO, CO_2_ or O_2_ molecule.

Normally, a material’s ideal (intrinsic) strength can be traced down to its bond strength^42^. The bond strength, in principle, can be greatly affected by the amount of charge in the bond. Removing one atom from the nanosheet will have a significant impact on the charge distribution of the layer, resulting in weakening the bond strength. As a result, the ideal strength of the material would be reduced. For example, the P-graphene is able to sustain an ideal strain above 30% (Fig. 2a), while the unavoidable defects in fabrication process (e.g. carbon atom vacancies) can significantly affect its strength, i.e. decreasing to only 14.8% (Fig. 3a). The introduction of foreign atoms in the defect sites of graphene brings new electron to interact with the dangling bonds, which therefore affect the bond strength and thus change the strain of the host materials. Motivated by this rationale, we explore the gas adsorption on D-graphene in order to modulate the ideal strain for D-graphene as well as shed new lights on the underlying mechanism.

Our computations show that the adsorption of H_2_, N_2_, CO, CO_2_ or O_2_ molecules makes the critical strain for D-graphene decrease by 0.4~2.0% (Fig. 3b). The most significant reduction comes from the CO or CO_2_ adsorbed D-graphene, where its breaking strain drops to only 12.8%. However, there is a shining exception: the NH_3_ dissociative adsorption enhances the ideal strain of D-graphene by approximately 1%, i.e. to 15.6%. Compared with the gas adsorption on the P-graphene, all the gas adsorptions turn to be energetically favourable on the D-graphene sheet (Table 2), and the remarkable high E_ad_ (9.083 eV) for NH_3_ also indicates that it dissociates on the D-graphene sheet. Furthermore, all of the six gas-adsorbed systems become magnetic.

**Figure 4:**
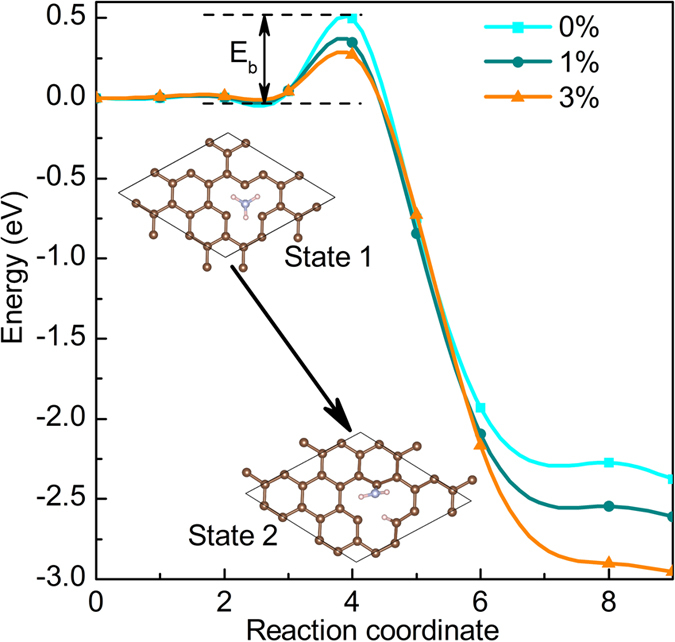
The energy barrier (*E*_a_) of NH_3_ dissociation on the defective graphene with the increase of strain.

With regard to the NH_3_ adsorption on defective graphene, experimentally it has been demonstrated that the NH_3_ molecule can easily dissociate on this host layer^43^. To validate the possibility of gas decomposition, we performed the nudged elastic band (NEB)^44^ DFT calculations for the NH_3_ + D-graphene system at different strains, as shown in Fig. 4. By computing the transition states, we find that the NH_3_ decomposition needs to overcome an energy barrier (*E*_a_) of 0.5 eV for the strain-free D-graphene, while the *E*_a_ value decreases as the strain increases. As a 0.5 eV energy barrier is not sufficient to hinder the NH_3_ decomposition, it is reasonable to expect the NH_3_ decomposition to occur without stain, while the finite strains can accelerate this process.

**Figure 5:**
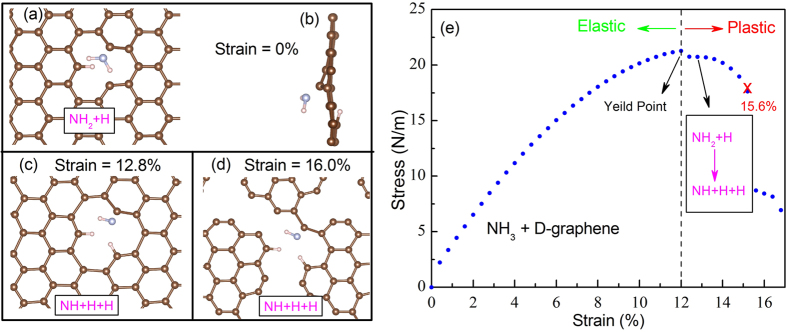
The structural configurations for the NH_3_ decomposition on the D-graphene sheet with (**a**,**b**) 0%, (**c**) 12.8% and (**d**) 16.0% strain. (**d**) The stress (σ) versus biaxial strain ε for NH_3_ adsorption in defective graphene.

The high possibility of NH_3_ decomposition on defective graphene, as revealed by our NEB calculations, is further verified by the optimized structural configuration of NH_3_ + D-graphene system (Fig. 5). The NH_3_ decomposition happens above the D-graphene sheet. When the strain is less than 12.8%, the NH_3_ molecule is dissociated into NH_2_ and H, NH_2_ is physisorbed at the carbon atom with a dangling bond, while H bonds to a neighbouring carbon atom (-c-H) (Fig. 5a–c). This dissociative NH_3_ adsorption induces obvious corrugation on the D-graphene layer (Fig. 5b) due to the strong electron coupling between the adsorbed species and the D-graphene sheet.

Interestingly, when the D-graphene is further stretched with a strain greater than 12.8%, the NH_2_ is further dissolved into NH and -c-H (Fig. 5d). Note that the H fragment plays a dominant role in saturating the dangling bond at the defect site, which would affect the charge distribution and strengthen the carbon bond (the neighbouring bond length reduces to 1.40 Å). As a result, the ideal strain for D-graphene is enhanced by the dissociative adsorption of NH_3_. Furthermore, we have also checked the structure for H_2_/N_2_/CO/CO_2_/O_2_ + D-graphene system, but no gas decomposition is found. Thus, the experimentally observed difference on the mechanical strength can be attributed to the differing impact of different gas adsorption at the defected site of graphene.

Finally, we examine the relationship between the stress and the strain for the NH_3_ + D-graphene system (Fig. 5e). The yield point of NH_3_ adsorbed graphene is at 12% strain. Before this point, it is in the elastic range where the deformation is reversible and the stretched layer can return to its original geometry when the tension is released. Further extension after the yield point would induce an irreversible plastic deformation, and the 2D structure will eventually rupture (see the structural change in Fig. 5d) after reaching the critical breaking strain of 15.6%.

## Conclusion

We systematically investigated the ideal strength for P-/D-graphene adsorbed by several common gas molecules including H_2_, N_2_, NH_3_, CO, CO_2_ and O_2_ by means of DFT computations. All the gas adsorption on P-graphene can significantly reduce its breaking strain, and the similar trend is also revealed for D-graphene that adsorbed by H_2_, N_2_, CO, CO_2_ and O_2_. Most importantly, the NH_3_ adsorption on D-graphene can significantly enhance its ideal strain to 15.6%, and the fundamental mechanism has been analysed in terms of NH_3_ dissociation. These results supply useful information for the ideal strength of gas adsorbed graphene system, and provide guidance to the fabrication of graphene-based electromechanical devices.

## Additional Information

**How to cite this article**: Ma, F. *et al*. Anomalous Enhancement of Mechanical Properties in the Ammonia Adsorbed Defective Graphene. *Sci. Rep.* **6**, 33810; doi: 10.1038/srep33810 (2016).

## Figures and Tables

**Table 1:** Calculated adsorbent-graphene distance (d), adsorption energy (E_ad_) and magnetic moment (M) of the gas molecule adsorbed perfect graphene layer in the equilibrium condition (0% strain).

	H_2_	N_2_	NH_3_	CO	CO_2_	O_2_
d (Å)	2.68	3.26	2.84	3.32	3.28	3.16
E_ad_ (meV)	−3.0	9.6	−4.0	0.7	3.3	−177
M (μ_B_)	—	—	—	—	—	1.915

**Table 2:** Calculated adsorbent-graphene distance (d), adsorption energy (E_ad_) and magnetic moment (M) of the gas molecule adsorbed D-graphene in the equilibrium condition.

	H_2_	N_2_	NH_3_	CO	CO_2_	O_2_
d (Å)	2.52	3.30	—	3.10	2.86	2.9
E_ad_ (meV)	−9.8	−201	−9083	−9.4	−28	−34.5
M (μ_B_)	0.23	0.27	1.41	0.24	0.22	2.19
